# Are Reports of Randomized Controlled Trials Improving over Time? A Systematic Review of 284 Articles Published in High-Impact General and Specialized Medical Journals

**DOI:** 10.1371/journal.pone.0084779

**Published:** 2013-12-31

**Authors:** Matthew J. To, Jennifer Jones, Mohamed Emara, Alejandro R. Jadad

**Affiliations:** 1 Electronic Living Laboratory for Interdisciplinary Cancer Survivorship Research, Princess Margaret Hospital, University Health Network, Toronto, Ontario, Canada; 2 Centre for Global eHealth Innovation, Princess Margaret Hospital, University Health Network, Toronto, Ontario, Canada; 3 Radiation Medicine Program, Princess Margaret Hospital, University Health Network, Toronto, Ontario, Canada; 4 Dalla Lana School of Public Health, University of Toronto, Toronto, Ontario, Canada; 5 Department of Anesthesia, Health Policy, University of Toronto, Toronto, Ontario, Canada; 6 Department of Management and Evaluation, University of Toronto, Toronto, Ontario, Canada; 7 Department of Psychiatry, University of Toronto, Toronto, Ontario, Canada; Université de Montréal, Canada

## Abstract

**Background:**

Inadequate reporting undermines findings of randomized controlled trials (RCTs). This study assessed and compared articles published in high-impact general medical and specialized journals.

**Methods:**

Reports of RCTs published in high-impact general and specialized medical journals were identified through a search of MEDLINE from January to March of 1995, 2000, 2005, and 2010. Articles that provided original data on adult patients diagnosed with chronic conditions were included in the study. Data on trial characteristics, reporting of allocation concealment, quality score, and the presence of a trial flow diagram were extracted independently by two reviewers, and discrepancies were resolved by consensus or independent adjudication. Descriptive statistics were used for quantitative variables. Comparisons between general medical and specialized journals, and trends over time were performed using Chi-square tests.

**Results:**

Reports of 284 trials were analyzed. There was a significantly higher proportion of RCTs published with adequate reporting of allocation concealment (p = 0.003), presentation of a trial flow diagram (p<0.0001) and high quality scores (p = 0.038) over time. Trials published in general medical journals had higher quality scores than those in specialized journals (p = 0.001), reported adequate allocation concealment more often (p = 0.013), and presented a trial flow diagram more often (p<0.001).

**Interpretation:**

We found significant improvements in reporting quality of RCTs published in high-impact factor journals over the last fifteen years. These improvements are likely attributed to concerted international efforts to improve reporting quality such as CONSORT. There is still much room for improvement, especially among specialized journals.

## Introduction

Since its publication in 1996, the Consolidated Standards of Reporting Trials (CONSORT) statement has been endorsed by over 400 journals worldwide, is available in seven different languages, and is viewed through its website more than 100,000 times each year. [1], [2].

Despite this high level of endorsement and dissemination, studies have suggested that inadequate reporting is still highly prevalent, even among journals that have endorsed the CONSORT statement calling for the evaluation of the impact of such reporting guidelines over a long period of time. [3], [4] Research also suggests that reporting quality varies according to the type of journal, with RCTs published in general journals having higher reported quality than those in specialized journals. [5] This study was designed to assess the quality of reports of RCTs evaluating interventions for chronic diseases that were published from 1995 to 2010 in high-impact journals that claim to have adopted the CONSORT statement.

The study only focuses on the quality of the reports of RCTs, not the quality of the RCTs as a whole, because the latter is likely an impossible undertaking. Quality, as beauty, anxiety, happiness, or love, is in the eye of the beholder, and means different things to different people, at different times, with different needs. [6] Therefore, this article provides data on key variables that have been empirically associated with an increased likelihood of bias in the original research effort, which could be easily reported by authors, and which could be easily identified as present or absent by readers.

## Methods

### Information Sources

The sample was drawn from a study designed to establish the frequency with which people living with multiple chronic diseases are excluded from RCTs published in high citation impact journals [7].

The trials were identified through MEDLINE and reports were included in the review if they:

- Described RCTs published from January to March of 1995, 2000, 2005, and 2010 in the five highest-impact factor general medical journals (*BMJ, CMAJ, JAMA, Lancet, and NEJM*) and specialized journals *(American Journal of Respiratory and Critical Care Medicine, Archives of General Psychiatry, Circulation, Diabetes,* and *Journal of Clinical Oncology)*, and
- Provided original data on the effects of interventions for chronic conditions (any incurable or long-lasting condition) in adults.

Trials on pediatric populations, post-trial follow-up studies, or secondary sub-group analyses were excluded.

### Data Extraction

Data extraction forms were piloted by members of the research team. Data were extracted and coded by two independent observers on the following variables: title; publication date; first author’s surname; country where the study was performed or coordinated (if multicenter); study design; and description of allocation concealment. Quality scores using the Jadad Scale were calculated from its individual items (descriptions of randomization, blinding, withdrawals and dropouts in RCT reports). As trials that were not randomized were excluded, scores of 1 to 2 out of 5 were regarded as indicating low quality. [8] Data were also obtained on whether the trial presented a participant flow diagram. Discrepancies were resolved by consensus by a third person whenever necessary.

### Data Synthesis

Descriptive statistics were used to summarize all quantitative variables. Comparisons between general medical and specialized journals, and trends over time were performed using Chi-square tests. All statistical tests were two-tailed and a p value lower than 0.05 was considered as statistically significant.

## Results

**Figure 1:**
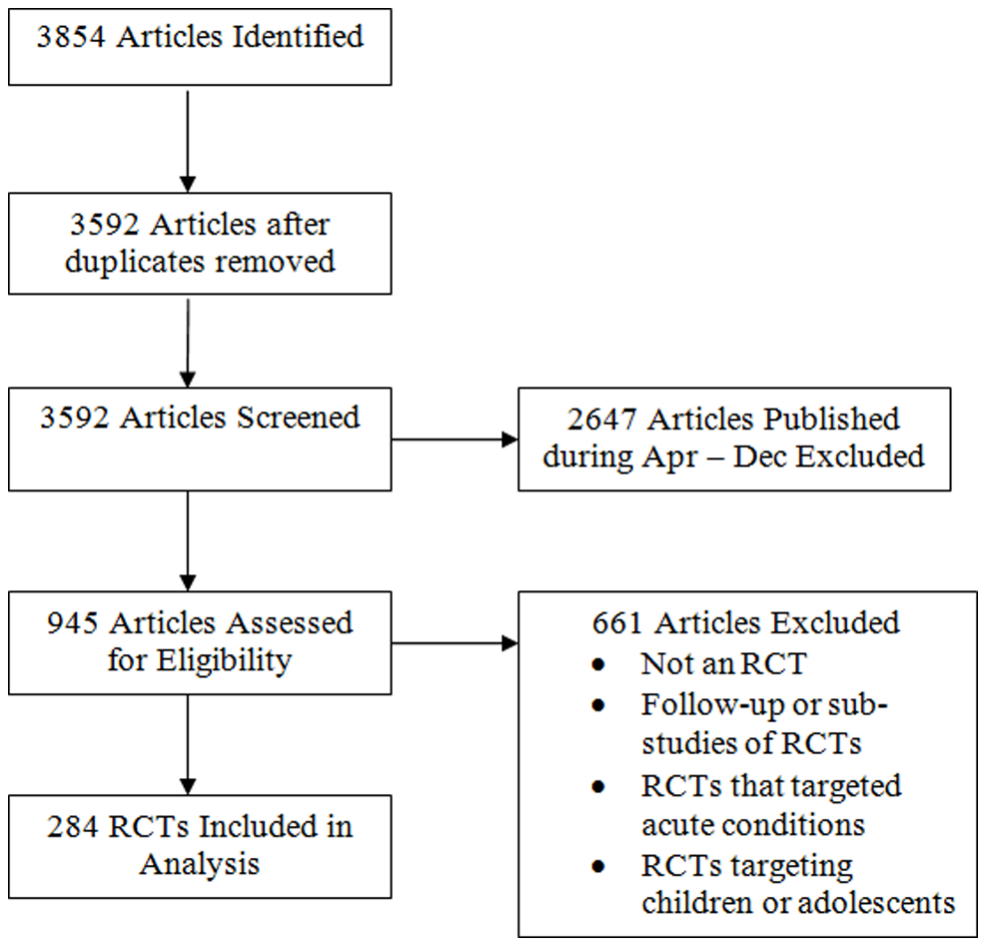
Flow Diagram of articles identified, screened, and included in analysis.

The search yielded 3854 potentially eligible articles. After screening their titles and abstracts, 284 reports were selected for inclusion in the analysis (Figure 1).

**Figure 2:**
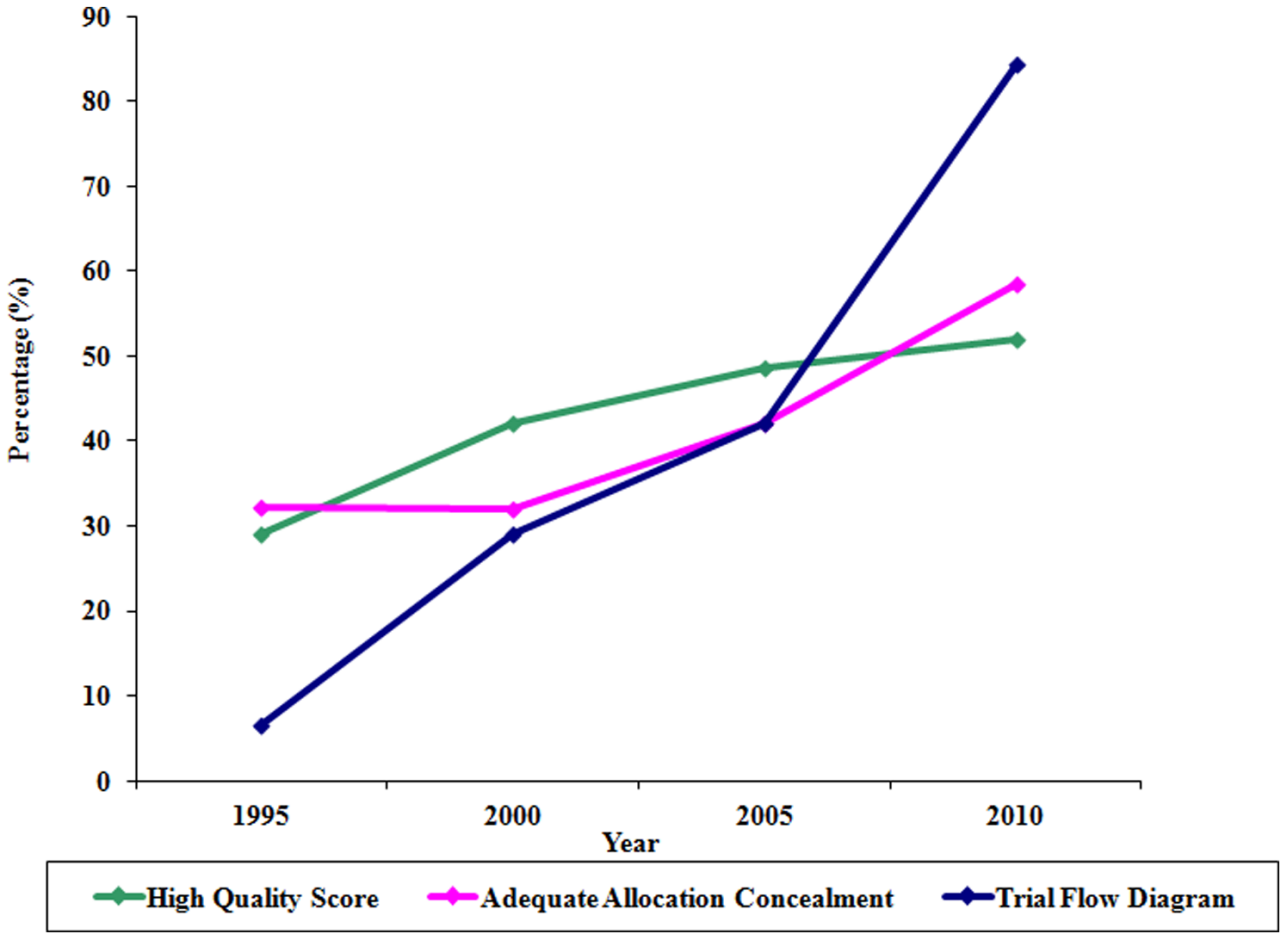
Quality scores, reporting of allocation concealment, and inclusion of trial flow diagram significantly improved over time.

Of those included, 165 of the trials were published in specialized journals (58.1%). Most of the studies used parallel study design (88.4%) and were conducted or coordinated in North America (48.2%) or Europe (46.5%). The majority of the reports (56.3%) received quality scores compatible with low methodological quality. Trials published in general medical journals had higher quality scores than those in specialized journals (55.5% vs. 35.2% had high quality scores, respectively, with p = 0.001), reported adequate concealment of allocation more often (50.4% vs. 35.8%, p = 0.013), and included a trial participant flow diagram more often (57.1% vs. 32.1%, p<0.001) (Table 1). There was a significantly higher proportion of reports with high quality scores over time (29% in 1995, 42% in 2000, 48.7% in 2005, 51.9% in 2010, p = 0.038) with the largest improvement occurring between 1995 and 2000. There were significant improvements in reporting of allocation concealment (32.3% in 1995, 31.9% in 2000, 42.1% in 2005, and 58.4% in 2010, p = 0.003) and presentation of a trial flow diagram (6.5% in 1995, 29.0% in 2000, 42.1% in 2005, and 84.4% in 2010, p<0.001) with the largest improvements occurring between 2005 and 2010 (Figure 2). Upon further analysis, there was a significantly higher proportion of reports published in general medical journals with high quality scores over time (21.9% in 1995, 56.0% in 2000, 67.9% in 2005, and 76.5% in 2010, p<0.001) while no significant differences in the proportion of reports published in specialized journals with high quality scores were observed over time (36.7% in 1995, 34.1% in 2000, 37.5% in 2005, and 32.6% in 2010, p = 0.961) (Table 2). Although reporting of allocation concealment in general medical journals improved over time, these differences were not significant (34.4% in 1995, 48.0% in 2000, 53.6% in 2005, and 64.7% in 2010, p = 0.100). Reporting of allocation concealment in specialized journals significantly improved over time (30.0% in 1995, 22.7% in 2000, 35.4% in 2005, 53.5% in 2010, p = 0.023). The inclusion of trial flow diagrams improved significantly over time for general medical journals (6.3% in 1995, 64.0% in 2000, 75.0% in 2005, 85.3% in 2010, p<0.001) and specialized journals (6.7% in 1995, 9.1% in 2000, 22.9% in 2005, 83.7% in 2010, p<0.001).

**Table 1:** Report Characteristics by Type of Journal.

	General Medical Journal(N = 119) No. (%)		Specialized Journal(N = 165) No. (%)		*X^2^* Value	P Value
Quality Score					13.558	0.009
1	21	(17.6)	44	(26.7)		
2	32	(26.9)	63	(38.2)		
3	40	(33.6)	37	(2.4)		
4	15	(12.6)	16	(9.7)		
5	11	(9.2)	5	(3.0)		
Low or High Quality Score					11.596	0.001
Low	53	(44.5)	107	(64.8)		
High	66	(55.5)	58	(35.2)		
Allocation Concealment					6.106	0.013
Adequate	60	(50.4)	59	(35.8)		
Inadequate	59	(49.6)	106	(64.2)		
Trial Flow Diagram					17.701	<0.001
Yes	68	(57.1)	53	(32.1)		
No	51	(42.9)	112	(67.9)		

**Table 2:** Report Characteristics by Type of Journal Over Time.

	1995 (N = 62)				2000 (N = 69)				2005 (N = 76)				2010 (N = 77)				*X^2^* Value	P Value
	General		Specialized		General		Specialized		General		Specialized		General		Specialized			
	No.	(%)	No.	(%)	No.	(%)	No.	(%)	No.	(%)	No.	(%)	No.	(%)	No.	(%)		
Quality Score																		
High	7	(21.9)	11	(36.7)	14	(56.0)	15	(34.1)	19	(67.9)	18	(37.5)	26	(76.5)	14	(32.6)	22.433*	<0.001*
Low	25	(78.1)	19	(63.3)	11	(44.0)	29	(65.9)	9	(32.1)	30	(62.5)	8	(23.5)	29	(67.4)	0.295**	0.961**
Allocation Concealment																		
Adequate	11	(34.4)	9	(30.0)	12	(48.0)	10	(22.7)	15	(53.6)	17	(35.4)	22	(64.7)	23	(53.5)	6.241*	0.100*
Inadequate	21	(65.6)	21	(70.0)	13	(52.0)	34	(77.3)	13	(46.4)	31	(64.6)	12	(35.3)	20	(46.5)	9.572**	0.023**
Trial Flow Diagram																		
Yes	2	(6.3)	2	(6.7)	16	(64.0)	4	(9.1)	21	(75.0)	11	(22.9)	29	(85.3)	36	(83.7)	48.972*	<0.001*
No	30	(93.8)	28	(93.3)	9	(36.0)	40	(90.9)	7	(25.0)	37	(77.1)	5	(14.7)	7	(16.3)	73.993**	<0.001**

Note: *denotes comparisons between all time points for variable in general medical journals; **denotes comparisons between all time points for variable in specialized journals

## Discussion

In this review, we present the results of one of the largest efforts to date to assess the reported quality of RCTs since the publication of the CONSORT statement. Our findings suggest that reporting quality of RCTs in the highest impact journals has improved significantly over the last fifteen years. The largest improvement was seen in the 13-fold jump in inclusion of a trial participant flow diagram (from 6.5% in 1995 to 84.4% in 2010) which could be attributed to the endorsement of the CONSORT diagram by most of the high impact journals included in the study and the development of international initiatives to improve reporting quality such as EQUATOR. [9] Although significant improvements in the number of trials with description of allocation concealment and with high quality scores were also observed over time, these two variables displayed different trends. Adequate description of allocation concealment improved considerably over the fifteen years with steady improvements at each successive time point, exceeding in 2010 what has been previously reported. [10], [11] Although an increasing trend of high-quality trials was observed over time, this increase appears to be leveling off with time, as there was only a 3% increase in the proportion of high-quality reports published between 2005 and 2010.

Although this study only examined selected items from reports of RCTs on interventions for chronic diseases published in high-impact journals, the findings appear consistent with previous research, suggesting that there have been improvements in reporting quality of RCTs in the biomedical literature as a whole, [12], [13] with RCTs published in CONSORT endorsing journals having relatively higher reported quality than reports published in non-CONSORT endorsing journals. [14] However, despite this encouraging trend, significant differences remain between general and specialized journals, with ample room for improvement, even among the former. Our findings are consistent with previous research showing that over half of the articles published in high-impact general medical journals reported adequate allocation concealment, [15] or that reports published in the specialist literature have shown little or no improvement since the development of the CONSORT statement. [16]–[19] Indeed, even those specialized journals that claim to support the CONSORT statement tend to comply less often than their general medical journal counterparts [5].

As this study was limited to assessment of several selected reporting items, additional analyses of various components of reported quality would yield more insights into the shortcomings of reporting of trials.

Poor compliance with reporting standards by authors and journal editors, just as poor adherence to therapies by patients or to clinical practice guidelines by clinicians, is becoming a chronic challenge for which effective interventions are urgently needed. Reporting guidelines such as the updated CONSORT statement, [20], [21] and the inclusion of clearer and stronger instructions to authors by journals [22] may not be sufficient.

It is important to underscore, however, that even if the reports of RCTs were perfect, such reports should not be equated with the quality of the trials themselves. There is no such thing as a perfect trial. As stated by Jadad and Enkin, [6] “To be perfect, among other things, trials would be designed in a way that would balance out all possible competing interests, and help to answer clear, relevant, previously unanswered questions. They would be conducted, and reported by researchers who did not have conflicts of interest, and who follow strict ethical principles.

They would have to evaluate all possible interventions, for all possible variations of the conditions of interest, in all possible types of patients, in all settings, using all relevant outcome measures. Moreover, they would include all available patients, and make full use of strategies to eliminate bias during the administration of the interventions, the evaluation of the outcomes, and reporting of the results, thus reflecting the true effect of the interventions.

The data would be properly analyzed statistically, and would include individual patient data, and an accurate description of the patients who were included, excluded, withdrawn, and who dropped out. The reports, which would give an exact account of all the events that occurred during the design and course of the trial, would be written in clear and unambiguous language.

Unfortunately, a perfect trial can only exist in our imagination. In real life, researchers can only do the best that they can, and report it as clearly as they can.”

## Conclusion

We found significant improvements in reporting quality of RCTs published in high-impact factor general and specialized journals over the last fifteen years. These improvements are likely attributed to concerted international efforts to improve reporting quality such as the CONSORT Statement. However, there is still much room for improvement, especially among specialized journals.

### Ethics Committee Approval

REB approval was not required or obtained for this study as it was a review of published articles and did not involve human subjects.

## Supporting Information

Checklist S1 (DOC)
